# Mechanisms involved in anti-aging effects of guarana (*Paullinia cupana*) in *Caenorhabditis elegans*

**DOI:** 10.1590/1414-431X20187552

**Published:** 2018-07-02

**Authors:** L.P. Arantes, M.L. Machado, D.C. Zamberlan, T.L. da Silveira, T.C. da Silva, I.B.M. da Cruz, E.E. Ribeiro, M. Aschner, F.A.A. Soares

**Affiliations:** 1 Departamento de Bioquímica e Biologia Molecular, Universidade Federal de Santa Maria, Santa Maria, RS, Brasil; 2 Universidade Aberta da Terceira Idade, Universidade do Estado do Amazonas, Manaus, AM, Brasil; 3 Department of Molecular Pharmacology, Albert Einstein College of Medicine, Bronx, NY, USA

**Keywords:** Aging, Antioxidant, Guarana, Lifespan, Natural products, Xanthines

## Abstract

Guarana *(Paullinia cupana)* is habitually ingested by people in the Amazon region and is a key ingredient in various energy drinks consumed worldwide. Extension in longevity and low prevalence of chronic age-related diseases have been associated to habitual intake of guarana. Anti-aging potential of guarana was also demonstrated in *Caenorhabditis elegans*; however, the mechanisms involved in its effects are not clear. Herein, we investigated the putative pathways that regulate the effects of guarana ethanolic extract (GEE) on lifespan using *C. elegans.* The major known longevity pathways were analyzed through mutant worms and RT-qPCR assay (DAF-2, DAF-16, SKN-1, SIR-2.1, HSF-1). The possible involvement of purinergic signaling was also investigated. This study demonstrated that GEE acts through antioxidant activity, DAF-16, HSF-1, and SKN-1 pathways, and human adenosine receptor ortholog (ADOR-1) to extend lifespan. GEE also downregulated *skn-1*, *daf-16*, *sir-2.1* and *hsp-16.2* in 9-day-old *C. elegans,* which might reflect less need to activate these protective genes due to direct antioxidant effects. Our results contribute to the comprehension of guarana effects *in vivo*, which might be helpful to prevent or treat aging-associated disorders, and also suggest purinergic signaling as a plausible therapeutic target for longevity studies.

## Introduction

*Paullinia cupana*, also referred to as guarana, is a native plant to the Amazon basin and especially common in Brazil. The powder of its seeds is habitually ingested by people of all ages in the Amazon region mainly for its tonic and stimulant properties (1). Moreover, guarana is a key ingredient in various energy drinks consumed in many countries (2). Other reported pharmacological effects of guarana include weight loss, lowering platelet thromboxane synthesis, protecting against gastric lesions, antioxidant activity, and anti-inflammatory effects [for review see: (1)]. However, when consumed in excess, guarana may also adversely affect human health, causing anxiety, sleep disruption, and tachycardia, for example, due to its high content of caffeine (2,3).

Extension in longevity in people living in Maués, an Amazon region in Brazil, has been associated to Amazonian diet, including habitual intake of guarana (4). Furthermore, an epidemiological study associated guarana ingestion with low prevalence of chronic age-related diseases in the Amazonian population (5). Recently, a study also demonstrated anti-aging potential of guarana seed extract in *Caenorhabditis elegans* (6). However, the mechanisms underlying the guarana effects on aging were not identified.

*C. elegans* has been a suitable model for understanding organismal responses to various synthetic and natural compounds and their influence on aging and lifespan. Vital biological pathways and numerous aspects of aging are analogous in nematodes and mammals, including humans (7).

Since the percentage of older people is growing worldwide accompanied with increased frequency of age-related disease, it is essential to identify efficacious therapies and therapeutic targets that might improve the quality of life (8). Herein, we investigated the putative pathways that regulate the effects of guarana on lifespan using *C. elegans.*

## Material and Methods

### Chemicals

Agar, ethanol (96%), chloroform, cholesterol, FUDR (5-fluoro-2′-deoxyuridine), protease inhibitor, phosphatase inhibitor cocktails, polymerase chain reaction (PCR) primers, and bovine serum albumin (BSA) were purchased from Sigma (USA). TaqMan¯ primers used for qRT-PCR analysis and Trizol were purchased from Applied Biosystems/Thermo Fisher Scientific Corporation (USA). All other reagents were purchased from Synth (Brazil).

### Strains and maintenance

Strains used in this study were Bristol N2 (wild-type); CB1370, *daf-2 (e1370) III*; CF1038, *daf-16 (mu86) I*; EU1, *skn-1(zu67)*; PS3551, *hsf-1(sy441) I*; TK22, *mev-1(kn1)*; and VC199, *sir-2.1(ok434)*, obtained from the *Caenorhabditis* Genetics Center (CGC), University of Minnesota, USA, as well as the *Escherichia coli* OP50. EG6890 strain, *ador-1 (ox489)*, was kindly supplied from Dr. Erik Jorgensen laboratory (University of Utah, USA). This strain has a deletion from 1 kb upstream and the first three exons of the *ador-1* gene, and was outcrossed 6 times; *ador-1* gene encodes an ortholog of human adenosine receptor (9).

Nematodes were maintained and assayed at 20°C on nematode growth medium (NGM) agar plates carrying a lawn of *E. coli* OP50 (10). Synchronization of nematode cultures was achieved by bleaching treatment of gravid hermaphrodites and eggs were allowed to hatch overnight in M9 buffer (42 mM Na_2_HPO_4_, 22 mM KH_2_PO_4_, 8.5 mM NaCl, and 1 mM MgSO_4_) (10).

### Plant material and extract preparation

The powder of toasted seeds of *Paullinia cupana* Kunth var. *sorbilis* (Mart.), the guarana, was isolated and supplied by EMBRAPA Oriental (Agropecuary Research Brazilian Enterprise) located in Western Amazon in Maués, Amazonas, Brazil. The hydro-alcoholic extract was obtained as described elsewhere (11). Briefly, the extract was produced using 70% ethanol. After 24 h, the resulting solution was filtered, the ethanol was removed, and the extract was lyophilized. The predominant xanthines and catechins presented in the guarana extract were analyzed by means of HPLC, showing the following concentrations: caffeine=12.240 mg/g, theobromine=6.733 mg/g and total catechins=4.336 mg/g (11).

### Treatment of the worms

NGM plates carrying a lawn of *E. coli* OP50 (as food source) were previously incubated at 37°C overnight. Lyophilized guarana ethanolic extract (GEE) was dissolved in cold distilled autoclaved water (121°C, 30 min) and spread over the plates at final concentrations of 100, 500 and 1,000 µg/mL of agar. Synchronized L1 larvae (10) were transferred with a pipette to the surface of treatment plates and cultured to adulthood at 20°C.

### Lifespan

Lifespan analyses started at L4 larvae in NGM plates seeded with *E. coli* OP50 in the absence or presence of GEE (day 0). Animals were transferred to fresh plates with or without GEE every other day to avoid confounding of generations, and scored at the same time until death. Absence of response to a mechanical stimulus was scored as death. Worms were censored if they crawled off the plate, displayed extruded internal organs, or died because of hatching progeny inside the uterus. Lifespan assays were repeated three times with 60–120 worms per assay. Through mutant strains, the major known longevity pathways were analyzed (12,13): i) *daf-2* and *daf-16*, the insulin/insulin-like growth factor (IGF)-1 signaling (IIS), which the DAF-2 receptor signals through a conserved PI3-kinase/AKT pathway and down regulates DAF-16/FOXO, responsible for promoting expression of genes that confer extended longevity and enhanced stress resistance; ii) *skn-1*, which is related to vertebrate Nrf family proteins and promotes expression of detoxification enzymes in response to oxidative stress, like glutathione-S-transferase; iii) *sir-2.1*, which encodes a histone deacetylase-like protein that integrates metabolic status with lifespan, and is associated to caloric restriction; and iv) *hsf-1*, which encodes heat-shock transcription factor-1 (HSF-1) and induces activation of various heat-shock genes or chaperones involved in maintaining the conformational homeostasis of proteins among other important functions. A possible relationship between longevity and purinergic signaling was also investigated through *ador-1* mutant strain.

As bacteria play a role in *C. elegans* mortality, *E.coli* OP50 growth was evaluated in the presence or absence of GEE to investigate if beneficial effects could be a response to an antimicrobial property. The absorbance of the bacteria was measured during a 12-h period in liquid medium (14).

### Health span

Behavior parameters related to health span were evaluated (15). Pharyngeal pumping was assessed with a Nikon E200 microscope by observing the number of pharyngeal contractions during a 60-s interval in wild-type young adults.

Thrash frequency was selected for analysis of locomotion. Wild-type young adults from control or GEE treatments were individually picked and placed in a drop of M9. The worms were allowed to adapt for 1 min and then the number of thrashes were quantified with a Nikon E200 microscope during a 20-s interval. A thrash was defined as a change in the direction of bending at the middle of the body.

Analyses were carried out in three independent assays. Thirty nematodes were examined per group.

### RNA isolation and real-time polymerase chain reaction (RT-qPCR)

Wild-type worms in 9-day-old adult worms were analyzed for gene expression related to longevity and oxidative stress responses. After adulthood, worms were transferred every other day to plates containing 150 mM of FUDR (5-fluoro-2′-deoxyuridine) to inhibit reproduction, in the presence or absence of GEE. RNA from 20,000 worms per condition was isolated using Trizol followed by chloroform extraction, as previously described (16) and 1 μg of input RNA was reverse transcribed to cDNA by Applied Biosystems high capacity cDNA reverse transcription kit (Applied Biosystems, USA). Expression analysis was performed by Custom TaqMan¯ Array analysis utilizing the corresponding TaqMan¯ Gene Expression assays for mitochondrial superoxide dismutase *sod-3* (Ce02404515_g1), glutathione-S-transferase *gst-4* (Ce02458730_g1), gamma glutamylcysteine synthetase *gcs-1* (Ce02436726_g1), *daf-16* (Ce02422835_g1), *sir-2.1* (Ce02459018_g1), *hsf-1* (Ce02423758_m1), heat shock protein *hsp-16.2* (Ce02506738_s1), and *skn-1* (Ce02407445_g1) (Applied Biosystems). Target gene expression was normalized to the expression values of actin *afd-1* (Ce02414573_m1). The relative expression of each gene was determined by the 2^−ΔΔCt^ method (17) and data are reported as fold change in mRNA levels relative to *afd-1*. This experiment was carried out in three independent worm preparations, each in triplicate.

### Statistical analysis

Statistical analyses were performed using GraphPad Prism version 5 for Windows (GraphPad Software, USA). The results are reported as means±SD of at least three individual experiments. Student's *t*-test was used to compare pairs of groups, whereas a one or two-way ANOVA followed by Bonferroni's *post hoc* test was used to compare three or more groups. All survival curves were analyzed by the log-rank (Mantel-Cox) test. Statistical significance was determined as P<0.05.

## Results

In our study, control wild-type *C. elegans* had a mean lifespan of 11 days and maximum lifespan of 14 days. In media containing GEE, mean lifespan of wild-type worms was extended to 13 days at 100 µg/mL (18%) and to 15 days at 500 and 1,000 µg/mL (36%). Maximum lifespan was extended by an average of 28% at the three tested concentrations (Table 1). There was no difference in *E. coli* growth in the presence or absence of 1,000 µg/mL of GEE (data not shown).

**Table 1.** Lifespan of untreated and guarana ethanolic extract (GEE)-treated *C. elegans*.

Genotype	GEE (µg/mL)	Mean lifespan ± SD (days)	Maximum lifespan ± SD (days)
Bristol N2	0	11±1.73	14±1.81
	100	13±2.00*	17±1.91*
	500	15±1.15*	18±1.07*
	1,000	15±1.63*	18±1.33*
*mev-1*	0	9±1.02	12±1.22
	1,000	13±1.07*	15±1.55*
*daf-2*	0	23±1.70	31±1.37
	1,000	29±1.64*	36±1.38*
*daf-16*	0	11±1.53	13±1.28
	1,000	11±1.57	12±1.44
*skn-1*	0	9±1.21	11±1.23
	1,000	9±1.35	11±1.38
*hsf-1*	0	11±0.2	13±0.7
	1,000	11±0.6	13±0.7
*sir-2.1*	0	12±1.80	16±1.42
	1,000	14±2.10*	17±1.66
*ador-1*	0	13±1.74	17±1.62
	1,000	12±1.68	16±1.33

Lifespan assays were performed at 20°C. Maximum lifespan is represented as the mean lifespan of the longest living 10% of the worm population. Each experiment was repeated three times starting with at least 60 nematodes per group. Data are reported as mean±SD. *P<0.05 compared to the untreated group (Mantel-Cox log-rank test).

**Figure 1.**
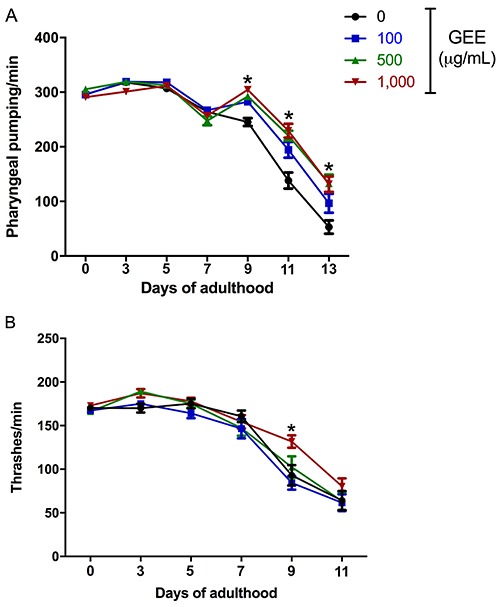
Guarana ethanolic extract (GEE) effects on behavioral parameters related to health span. Pharyngeal pumping rate (*A*) and thrash frequency (*B*) during aging in wild-type worms. Data are reported as mean±SD (n=30 worms per group). *P<0.05 compared to control (one-way ANOVA followed by Bonferroni multiple comparison test.

The health span of the worms was also prolonged after GEE treatment. The extract delayed the age-related decline in pharyngeal pumping (100, 500, and 1,000 µg/mL) and thrashes (1,000 µg/mL) starting on the 9^th^ day of adulthood (Figure 1 A and B). Accordingly, the concentration of 1,000 µg/mL of GEE and samples of 9-day adult worms were selected for further analysis.

GEE extract (1,000 µg/mL) extended mean lifespan of *mev-1* mutants by 44% and maximum lifespan by 77.8% (Table 1), showing a connection between antioxidant and anti-aging activities. The extract also extended mean lifespan of *daf-2*, and *sir 2.1* mutants, establishing that the extract did not act through these pathways to promote lifespan extension. In contrast, the treatment did not prolong lifespan of *daf-16, skn-1, hsf-1*, and *ador-1* mutants (Table 1).

PCR analyses assessed gene modulation by GEE (Table 2). GEE at 1,000 µg/mL down regulated *skn-1*, *daf-16*, *sir-2.1*, and *hsp-16.2* in 9-day-old adults. No effect was observed on *hsf-1*, *gst-4*, *gcs-1*, and *sod-3* expression.

**Table 2.** Fold change mRNA expression of genes related to longevity and oxidative stress in wild-type 9-day-old adult worms treated with guarana ethanolic extract (GEE)

Gene	0 µg/mL GEE	1,000 µg/mL GEE
*skn-1*	−0.017±0.037	−0.613±0.133*
*daf-16*	−0.026±0.087	−0.692±0.133*
*sir-2.1*	+0.015±0.072	−0.708±0.164*
*hsf-1*	+0.001±0.104	−0.154±0.150
*gst-4*	+0.005±0.114	−0.083±0.125
*gcs-1*	+0.006±0.116	−0.059±0.193
*sod-3*	+0.005±0.117	−0.388±0.137
*hsp-16.2*	−0.010±0.089	−0.670±0.228*

This experiment was assessed by RT-qPCR and carried out in three independent worm preparations, each in triplicate. Data are reported as means of fold change in mRNA levels relative to *afd-1* (actin) ±SD. *P<0.05 compared to untreated (two-way ANOVA followed by Bonferroni's multiple comparison test).

## Discussion

As previously demonstrated (1), in our study, guarana extract also extended lifespan and health span of wild-type *C. elegans*. Thus, putative pathways that might be implicated in its anti-aging effects were investigated. Herein, anti-aging effects were shown at a higher concentration of the extract (1,000 *vs* 300 µg/mL as previously demonstrated). This discrepancy may be due to differences in extract preparation (hydro-alcoholic *vs* aqueous extract) and delivery method (agar *vs* liquid medium) and may be a result of natural drifting in genetic variation in the worms' population (18,19).

Oxidative stress appears to be a major factor limiting lifespan in both *C. elegans* and humans and is associated to many age-related diseases (20,21), which directs attention toward antioxidant compounds with effects *in vivo*. To further investigate whether GEE could extend lifespan through an antioxidant activity, its effect on *mev-1* worms was evaluated. This strain is characterized by superoxide overproduction and has a shorter lifespan compared to wild-type strain (22). Consistent with previously described antioxidant effects of guarana extract (6), GEE treatment significantly extended mean and maximum lifespan of *mev-1* worms.

Besides that, DAF-16, HSF-1, and SKN-1 pathways, involved in the insulin/IGF signaling (IIS), appeared essential for GEE-mediated lifespan extension. Reduced IIS is associated with longevity and adaptation to adverse environmental conditions in *C. elegans*, Drosophila, mammals, and possibly humans (13). HSF-1 functions in cooperation with DAF-16 to activate the expression of common target genes, including the family of *sHsp* (small heat shock proteins genes) (23). SKN-1/Nrf integrates IIS and regulates response to oxidative stress and expression of detoxification genes (24).

DAF-16, HSF-1, and SKN-1 might also mediate health span extension and protein homeostasis (25). DAF-16 is involved in the formation of less toxic high-molecular weight protein aggregates (26), and although HSF-1 regulates protein disaggregation activity releasing small toxic aggregates, it might have a beneficial effect contributing to protein clearance through enzymatic metabolism (27,28). SKN-1 is best known as a regulator of antioxidant and xenobiotic defense, but it has also been implicated in additional functions that include proteostasis and metabolic regulation (24).

Methylxanthines, as caffeine, are the main components of guarana and it is well known that these compounds can act through adenosine receptors in mammals (29). Caffeine has been associated with beneficial effects, including aging-related effects (30,31) and improvement of cognitive impairment phenotypes by antagonizing the adenosine receptors A_1_ and A_2A_ in rodents (32). Thus, we tested if the GEE-induced extension of lifespan might also depend upon ADOR-1, an adenosine receptor homolog (33). Our results indicated that *ador-1(ox489)* worms failed to show extended lifespan, demonstrating, for the first time, a possible role of the purinergic system in lifespan extension. Accordingly, purinergic signaling may be profitably studied in the future as a potential target for longevity modulation.

Although GEE has high levels of caffeine, and previous studies described caffeine's effects in worms (34–36), the anti-aging effects of GEE might be related to synergic effects of different compounds. The concentration of caffeine in the extract is much lower than the effective concentration previously demonstrated and it was shown that alkaloid extract from guarana did not have the same beneficial effects (6). Besides, data from the literature shows that extracts could have greater pharmacological activities than isolated compounds (37,38).

Downregulation of *skn-1*, *daf-16*, *sir-2.1*, and *hsp-16.2* in 9-day-old *C. elegans* treated with GEE might reflect less need to activate these genes to repair cell damage during aging compared to untreated worms, possible due to direct antioxidant effects exerted by the extract (39,40).

Thus, this study showed that anti-aging effects of guarana are mediated by antioxidant activity and DAF-16, HSF-1, and SKN-1 pathways. In addition, ADOR-1 was also necessary for GEE effects on lifespan, indicating a possible involvement of the purinergic system in longevity. Our results contribute to the comprehension of guarana effects *in vivo*, which might be helpful to prevent or treat aging-associated disorders, and suggest purinergic signaling as a plausible therapeutic target for longevity studies.
